# Development of ATAQ-LAM: a tool to assess quality of life in Lymphangioleiomyomatosis

**DOI:** 10.1186/s12955-015-0294-5

**Published:** 2015-07-30

**Authors:** Tarik D. Walker, Jennifer Desserich, Karen Albright, Frederick S. Wamboldt, Amanda Belkin, Kaitlin Fier, Jeffrey J. Swigris

**Affiliations:** Department of Pediatric Infectious Disease, University of Colorado School of Medicine, Aurora, CO USA; Autoimmune Lung Center and Interstitial Lung Disease Program, National Jewish Health, 1400 Jackson Street, Denver, CO 80206 USA; Department of Community and Behavioral Health, Colorado School of Public Health and Colorado Health Outcomes Program, University of Colorado School of Medicine, Aurora, CO USA; Division of Pulmonary and Critical Care Medicine, Sleep and Behavioral Health Sciences Section, National Jewish Health, Denver, CO USA

## Abstract

**Background:**

Lymphangioleiomyomatosis (LAM) is a progressive lung disease that impairs health-related quality of life (HRQL).

**Objective:**

To develop and conduct initial testing of ATAQ-LAM (A Tool to Assess Quality of Life in LAM).

**Methods:**

A pilot version of the questionnaire was administered to respondents with LAM. We used a deletion algorithm to retain items and then applied multi-trait scaling to place retained items into appropriate domains, thus generating the ATAQ-LAM. Rasch analysis was used to assess item fit to a unidimensional model of HRQL. We determined internal consistency (IC) and floor and ceiling effects of ATAQ-LAM scores and conducted analyses aimed at supporting the validity of ATAQ-LAM.

**Results:**

Sixty-nine LAM patients provided response data. Thirty-two items survived the deletion algorithm. Scaling suggested ATAQ-LAM should have a four-domain structure (Exertional dyspnea, IC = 0.94; Cough, IC = 0.91; Fatigue, IC = 0.91; Emotional Well-Being, IC = 0.89). All items fit the Rasch model. Among 17 respondents with spirometry within three months of questionnaire completion, three of five ATAQ-LAM scores correlated with FEV1% (Exertional Dyspnea: r = −0.72, p = 0.001; Fatigue: r = −0.62, p = 0.007 and total: r = −0.53, p = 0.02). Compared with those in the highest tertile of FEV1%, subjects in the lowest tertile had greater ATAQ-LAM total (121.8 ± 14.3 vs. 79.8 ± 13.1, p = 0.04), Exertional Dyspnea (54.4 ± 6.3 vs. 25.5 ± 5.8, p = 0.005) and Fatigue (2.8 ± 2.4 vs. 14.8 ± 2.3, p = 0.03) scores, indicating greater impairment in HRQL.

**Conclusions:**

ATAQ-LAM is a disease-specific instrument designed to assess HRQL in LAM patients. Additional studies are needed to generate data in support of its validity as an instrument capable of assessing HRQL over time in LAM patients.

**Electronic supplementary material:**

The online version of this article (doi:10.1186/s12955-015-0294-5) contains supplementary material, which is available to authorized users.

## Introduction

Lymphangioleiomyomatosis (LAM) is an incurable, low-grade malignancy that occurs either sporadically (S-LAM) or as a result of tuberous sclerosis complex (TSC-LAM). LAM affects predominantly women of child-bearing age, and although its rarity creates challenges in generating epidemiological estimates, LAM has been reported to occur in about 5 persons per 100,000 in the U.S [1–3]. In LAM, viable lung tissue is progressively replaced by thin-walled cysts, leading to an obstructive ventilatory defect and increasingly severe dyspnea [4].

Besides dyspnea, other intrusive symptoms include cough and fatigue. Given the symptoms, need for supplemental oxygen and the anxiety of living with a potentially life-shortening illness, it stands to reason that health-related quality of life (HRQL) may be impaired in patients living with LAM [5]. Recent advances in understanding the pathogenesis of LAM, including the identification of promising therapeutic targets, have paved the way for testing novel or existing drugs [6]. A trial (called MILES and designed to assess the efficacy and safety of rapamycin in LAM) showed that the drug halted LAM progression (as defined by decline in the one-second forced expiratory volume [FEV_1_]) and was associated with improvements in scores from one generic HRQL measure (the EuroQOL visual analogue scale for quality of life) but not other generic (the General Well-Being Questionnaire or the SF-36) or a chronic obstructive pulmonary disease specific instrument (the St. George’s Respiratory Questionnaire or SGRQ) [6]. Whether a LAM-specific HRQL questionnaire would have performed similarly (or better) is unknown, because one was not available for use at the time MILES was conducted; however, it is believed that carefully developed, patient-tailored, and disease-specific instruments are likely to be more sensitive to underlying change than generic instruments [7].

We previously conducted seven focus groups with 37 TSC- or S-LAM patients to better understand how LAM affects patients’ lives [5] and to gather data that we used to develop content (domains and items) for a preliminary version of a LAM specific HRQL instrument. Shortness of breath, fatigue and cough were the symptoms mentioned in all seven groups, but shortness of breath was by far the most bothersome. The psychological experiences of living with LAM included frustration with the physical limitations LAM imposes; worry over living with an unpredictable, incurable, life-threatening illness; and myriad issues associated with assuming the “patient role”. Such issues included a loss of identity, the perception of being viewed as weak when yielding to physical limitations, embarrassment when using supplemental oxygen, and the loss of control that came with needing to take medication to fight the disease.

Here, we describe the development and initial validity analyses of ATAQ-LAM (A Tool to Assess Quality of Life in LAM), a LAM-specific, multi-dimensional instrument to assess HRQL.

## Methods

### Phase I: Item pool development and evaluation

Guided by qualitative content analysis of the transcripts from the focus groups [5] and clinical experience caring for LAM patients, we developed a pool of 56 items comprising a preliminary version of ATAQ-LAM. The qualitative work revealed themes including physical manifestations of shortness of breath, fatigue, cough, chest sensations, difficulty sleeping, and GI issues along with the psychological manifestations mentioned above.

In developing the items, we elected to focus on the most “proximal” effects—those that can be most directly linked back to living with LAM. Thus, the pilot version of ATAQ-LAM included the generated items grouped into 10 hypothesized domains: Exertional dyspnea (14 items), Effects of dyspnea (4 items), Chest pain (2 items), Cough and wheeze (13), Fatigue (7 items), Emotional well-being (7 items), Relationships (2 items), Sexuality (2 items), Symptom-specific HRQL (4 items), and Global HRQL (1 item). The recall period was 48 h. Each item was structured identically: between two contrasting statements were six numerical response options (1, 2, 3, 4, 5, 6)—a response of “1” indicates the strongest agreement with the statement on the left, and a response of “6” indicates the strongest agreement with the statement on the right. Summation scoring was used for the total and each domain, with higher scores connoting greater impairment.

### Phase II: Item reduction and validation

#### Subjects and general overview

A convenience sample of subjects was recruited at the 2014 LAMposium in Chicago, Illinois. LAMposium is an annual, three-day conference that offers patients the opportunity to become educated about LAM and to connect with one another. After providing written, informed consent, each subject completed the pilot version once. Demographic and clinical data (including most recent percent predicted one-second forced expiratory volume [FEV1%] results, oxygen- and medication-use) were collected from patient records. After LAMposium, 11 LAM patients participated in debriefing interviews (Please see the Supplement for their baseline characteristics). All themes and items were deemed relevant by interviewees, and no other new topics or items were suggested, despite extensive probing.

#### Ethics, consent and permissions

All participants gave written, informed consent to participate. The study protocol was approved by the National Jewish Health Institutional Review Board (protocol #HS-2774).

#### Statistical approach

##### General

Summary statistics were generated for baseline characteristics of the study sample.

##### Item deletion

We began the analysis by passing items through an item-deletion algorithm. Please see the Supplement for full details. First, items for which fewer than five response options were used were deleted (N = 3). Next, items missing responses from greater than 20 % of the sample were deleted (N = 2). Surviving items with greater than 49 % of respondents scoring at the floor (N = 13) or ceiling (N = 0) were considered for deletion, one of which was retained. Other items (N = 7) were deleted for item-item correlations > 0.7. Lastly, after thoughtful consideration, two “relationship” items were deleted because of concerns over lack of specificity to the experience of living with LAM. Ultimately, 32 items in 8 domains were retained: Exertional dyspnea (8 items), Effects of dyspnea (4 items), Chest pain (1 item), Cough (5 items), Fatigue (4 items), Emotional well-being (5 items), Symptom-specific HRQL (4 items) and global HRQL (1 item). The lone Chest pain item was moved to the Symptom-specific HRQL domain (thus yielding 7 domains).

##### Item scaling

We began the examination of the 32 retained items with a multi-trait scaling analysis, which is a method to address internal consistency reliability, item convergence, and item discrimination [8]. This analysis generates a correlation matrix, with each row representing an item and each column a hypothesized domain. This matrix allows for evaluation of the extent to which an item correlates with its hypothesized domain. An item that correlates at a level of 0.3 or greater with its hypothesized domain possesses convergent validity, and an item whose correlation with its hypothesized domain is significantly greater than its correlation with any other domain possesses discriminant validity [8]. Meng and colleagues’ method [9] was used to test whether the difference between these correlation coefficients was statistically significant. With results from this analysis, items can be rescaled (moved from one domain to another, better-fitting, domain), thus yielding the most systematically-structured domains. At the end of this analysis, redistribution yielded four domains (Exertional dyspnea, Cough, Fatigue, and Emotional well-being) and three other items that contribute to a total score but do not belong to any domain. Please see the Additional file 1 for full details.

##### Rasch analysis

Next, we subjected all items in aggregate to Rasch analysis (Winsteps, Version 3.69.1.14, www.winsteps.com). The mathematics of the Rasch model locates items and patients on the same scale according to—for an item—the level of HRQL impairment it signifies and—for a patient—her level of HRQL impairment (as determined by her responses to all items) [10]. Rasch uses the difference between item and patient location—many terms have been used, but here we refer to these locations as item difficulty and patient severity, respectively—to model the probability of responses to each item. Because Rasch analysis is based on Guttman scaling, a patient’s endorsement of any item implies her endorsement of less difficult items (i.e., items of lesser difficulty or lower on the domain). Rasch analysis allows determination of whether a dataset adheres to fundamental measurement properties, an important one being unidimensionality—items function together to assess a singular construct (in the case of ATAQ-LAM, it would be LAM-specific HRQL). We assessed item fit by using the infit mean square statistic; values from 0.5-1.5 are considered useful for measurement, and values greater than 2.0 degrade measurement [11].

##### Performance characteristics and validity

Summation scoring was used for each domain and total. For each domain, we calculated mean scores, internal consistency reliability using Cronbach’s coefficient alpha, [12] and the percentage of respondents at their floor and ceiling values. In the “validity analyses,” we used the Pearson product–moment method to examine correlations between ATAQ-LAM scores and FEV1% (among patients who performed spirometry within three months of completing ATAQ-LAM). We hypothesized that scores from all but the cough domain would be significantly correlated with FEV1%. We also hypothesized that ATAQ-LAM scores would be greater (connoting more impaired HRQL) among patients with more severe LAM; we tested this hypothesis by conducting two analyses: 1) we used ANOVA to compare ATAQ-LAM scores among three subgroups defined by tertiles of FEV_1_%; and 2) we used t tests to compare ATAQ-LAM scores between subjects using—versus not using—supplemental oxygen.

Analyses other than Rasch were performed using SAS Version 9.3 software (SAS, Inc.; Cary, NC). We considered p < 0.05 to represent statistical significance.

## Results

The sample included 69 female subjects with a broad range of LAM duration (Table 1). Nearly half the subjects were taking rapamycin. Among the 17 subjects with useable spirometry data, mean FEV1% suggested moderate obstruction.

**Table 1:** Baseline characteristics of study participants

Variable	N	Results
Female		69
Age in years	69	49.1 ± 12.0 (range 24 – 73)
Race	68	
African-American		2
Asian		3
Hispanic		5
White		58
Education	65	
High-school		2
Some college		10
Trade school graduate		2
College graduate		28
Masters		18
Doctorate		5
Employment	64	
Full-time		26
Part-time		8
Retired		8
Disabled		14
Unemployed		8
Smoking history	67	
Current		0
Former		18
Never		49
LAM duration, years	69	8.2 ± 7.9 (range 0.03 – 40)
FEV1, L	17	1.70 ± 0.6 (range 0.79 – 2.87)
FEV1%	17	65.1 ± 22.5 (range 32 – 95)
Supplemental oxygen	67	
Never		38
Ever		29
Sleep only		2
Exertion only		9
Sleep + exertion, not at rest		12
All the time		6
Medications		
Combination CS/LABA		26
Doxycycline		1
Plaquenil		1
Rapamycin		30
Statin		18

*LAM* lymphangioleiomyomatosis, *FEV1%* percent predicted one-second forced expiratory volume, *CS/LABA* combination inhaled corticosteroid and long-acting beta-agonist

**Fig. 1:**
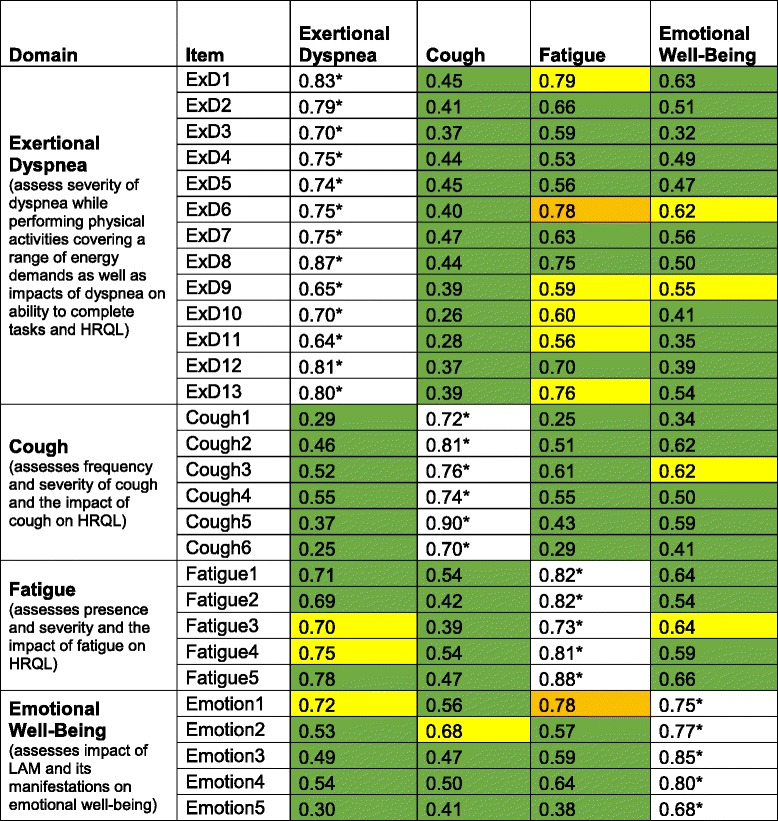
Multi-trait correlation matrix for 29 ATAQ-LAM items that form the Exertional Dyspnea, Cough, Fatigue and Emotional Well-Being subscales. Footnote: ExD=exertional dyspnea domain; each row is an item, and each column is a domain; values are correlations between an item and each of the four domains; white=items hypothesized to belong to the domain indicated by its column header; perfect scaling for an item would be indicated by a row with three green rectangles and one white rectangle that contains an asterisked value; *=correlation in white is significantly stronger than correlations in same row marked green (scaling success); yellow=probable scaling success—correlation coefficient in white is greater than correlation coefficient in yellow, but the difference did not reach statistical significance; orange=possible scaling success—correlation coefficient in white is lesser than correlation coefficient in orange, but the difference did not reach statistical significance

Figure 1 shows the multi-trait correlation matrix and heat map for the 29 items within the four domains. Eighty one (of 96) comparisons (shown in green) in the matrix were scaling successes (correlation between an item and its hypothesized domain was significantly stronger than the correlation between the item and any of its three non-hypothesized domains). For the remaining 15 comparisons, including the 2 where the correlation coefficient was greater in a non-hypothesized domain than the hypothesized domain (shown in orange), there was no statistical difference.

**Table 2:** Properties of four scales of ATAQ-LAM

Domain	alpha	Items	Mean correlation among items within the domain	Range possible	Range used	Mean ± SD^a^	% at floor	% at ceiling
Exertional dyspnea	0.94	13	0.75	13-78	13-73	33.9 ± 15.1	4.7	0.0
Cough	0.91	6	0.77	6-36	6-29	13.8 ± 7.1	15.6	0.0
Fatigue	0.91	5	0.81	5-30	5-29	15.4 ± 7.1	6.3	0.0
Emotional	0.89	5	0.77	5-30	5-30	14.3 ± 7.2	10.9	4.7
Total	0.96	32		32-192	35-151	84.6 ± 34.1	0.0	0.0

*ATAQ-LAM* A Tool to Assess Quality of life in Lymphangioleiomyomatosis, *Emotional* Emotional Well-Being domain, *alpha* Cronbach’s coefficient alpha

^a^higher scores indicate greater impairment

**Fig. 2:**
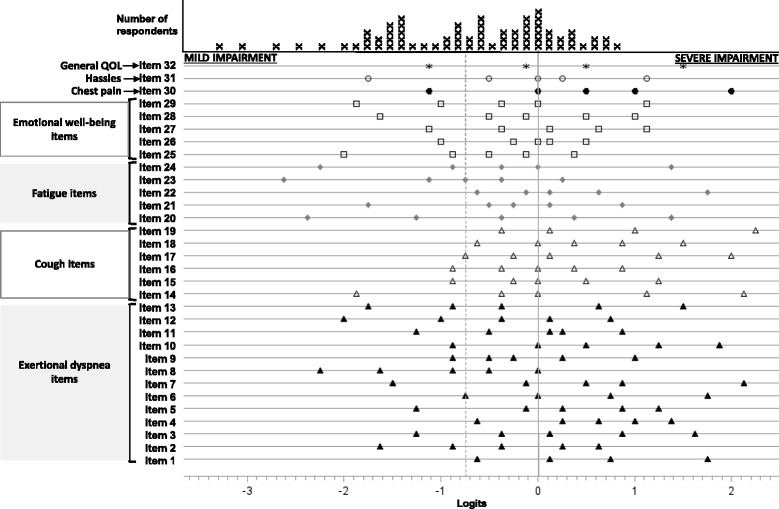
Boxplots of ATAQ-LAM total scores with the subgroup of subjects with useable data stratified on FEV1%. Footnote: Horizontal lines within the boxes represent the median. Diamonds represent the mean. Box ends represent the 25th and 75th quartiles. Whiskers represent two standard deviations from the main. The circle is an extreme value

All items fit the Rasch model. Figure 2 shows the positions of the thresholds between response options for each item along with other items in its domain. Table 2 shows some of the performance characteristics of the ATAQ-LAM domains and total. Please see the Supplement for a copy of ATAQ-LAM.

**Table 3:** Comparison of ATAQ-LAM scores between subjects using any—versus no—supplemental oxygen

Domain	Any O2	No O2	Difference, SDU	p
	N = 29	N = 35		
Exertional dyspnea	43.1 ± 14.0	27.7 ± 12.5	15.4 ± 13.1, 1.01	<0.0001
Cough	15.6 ± 6.7	12.5 ± 7.1	3.1 ± 6.9, 0.44	0.08
Fatigue	19.2 ± 7.2	12.7 ± 5.6	6.5 ± 6.3, 0.92	0.0001
Emotional	17.1 ± 8.5	12.3 ± 5.6	4.8 ± 6.9, 0.67	0.01
Total	104.1 ± 34.1	71.3 ± 27.3	32.7 ± 30.2, 0.96	<0.0001

*Emotional* Emotional Well-Being domain, *O2* supplemental oxygen, *SDU* standard deviation units for differences

**Fig. 3:**
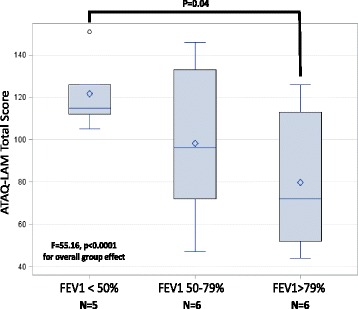
Item thresholds derived from Rasch analysis. Footnote: Items are situated on the vertical access and grouped into their respective domains. Note there are three items (30, 31, and 32) that are included in the ATAQ-LAM total score but do not contribute to any of the four domain scores. The small figures (circle, square, etc.) on the horizontal line extending from an item label represent threshold positions between response options for that item. Thresholds represent the logit position where a patient is equally likely to respond to either of two adjacent response options (1 or 2, 2 or 3, 3 or 4, 4 or 5, 5 or 6). For items with four thresholds, only five response options were used by the sample. The solid vertical line at “0” logits is the mean item difficulty. The dashed vertical line at −0.75 logits represents the mean patient severity. Each “X” along the top of the figure represents one respondent (LAM patient) and her HRQL severity location (in logits). HRQL impairment ranges from mild (left) to severe (right). Likewise, item difficulty ranges from easy (likely to be endorsed by someone with even mild HRQL impairment) on the left to difficult (likely to be endorsed only by someone with the greatest HRQL impairment) on the right

Three of five ATAQ-LAM scores correlated with FEV1% (Exertional Dyspnea: r = −0.72, p = 0.001; Cough: r = 0.05, p = 0.84; Fatigue: r = −0.62, p = 0.007; Emotional Well-being: r = −0.24, p = 0.34; total r = −0.53, p = 0.02). Compared to subjects within the highest tertile of FEV1% (values >79), those in the lowest tertile (values <50 %) had significantly greater scores for three domains: 1) ATAQ-LAM total (Fig. 3, 121.8 ± 14.3 vs. 79.8 ± 13.1, p = 0.04); 2) Exertional Dyspnea (54.4 ± 6.3 vs. 25.5 ± 5.8, p = 0.005) and 3) Fatigue (22.8 ± 2.4 vs. 14.8 ± 2.3, p = 0.03). But as hypothesized, there was no difference for Cough (16.2 ± 10.0 vs. 16.0 ± 6.1, p = 0.97). Subjects using supplemental oxygen had significantly higher mean ATAQ-LAM scores (all except for the Cough domain) than subjects who did not use supplemental oxygen (Table 3).

## Discussion

We developed ATAQ-LAM (“A Tool to Assess Quality of Life in LAM”), a 32-item questionnaire to assess HRQL in patients with LAM—a cystic lung disease with intrusive symptoms that affect how patients feel and function. Importantly, and in accordance with the movement to more-meaningfully include patients in the research enterprise, ATAQ-LAM was built by incorporating LAM patients’ perspectives at the ground level (in item generation). Through item deletion, we were left with items that cover the most prominent, important and proximal effects of LAM-related HRQL.

In 2002, the Scientific Advisory Committee (SAC) of the Medical Outcomes Trust [13] described standards for eight attributes that HRQL instruments should possess, including: 1) conceptual and measurement model; 2) reliability; 3) validity; 4) responsiveness; 5) interpretability; 6) respondent and administrative burden; 7) alternative forms; and 8) cultural and language adaptations. Although there is much more work to be done, the development strategy and analyses performed here confirm that certain psychometric properties—and by extension, certain SAC standards—of ATAQ-LAM are acceptable. The multi-trait analysis indicated that the final scaling should yield domains responsive to change over time and with the ability to discriminate between LAM patients with different degrees of HRQL impairment. Rasch analysis confirmed that overall, the items targeted the study sample well: there was good matching of items to the severity of HRQL impairment. Internal consistency reliability was excellent; the percent of subjects scoring at the minimum or maximum for any score suggests a very low possibility for significant floor or ceiling effects. Missingness was minimal. To calculate domain scores when responses are missing, we would suggest imputing responses for missing items using the mean from the non-missing items in a domain (assuming at least half the items in that domain have responses).

Although “validation” is never truly finished—rather, it is an ongoing process of testing hypotheses and gathering data—validity of ATAQ-LAM was supported by three findings: 1) significant correlation, in the expected direction, between ATAQ-LAM scores and FEV1%; 2) subjects requiring supplemental oxygen had higher ATAQ-LAM scores than subjects not requiring supplemental oxygen; and 3) ATAQ-LAM scores (total, Exertional Dyspnea and Fatigue) were significantly higher (more impaired HRQL) for subjects with the lowest FEV1% compared with subjects with the highest FEV1% values. The latter reveal that ATAQ-LAM can discriminate between patients with differing levels of LAM severity—and by extension, differing levels of HRQL impairment.

In a prior study, the SGRQ was observed to possess validity in LAM [14]. One can presume that by including items most relevant to patients with LAM (and excluding those that are not, including several items on the SGRQ), ATAQ-LAM would be more sensitive to change than SGRQ; whether this is true remains a question that requires further study. In the MILES Trial, rapamycin halted physiological decline in patients with LAM, but did not lead to significant improvements in EuroQOL dyspnea or fatigue scores. Although ATAQ-LAM covers dyspnea and fatigue, the items are distinct from the EuroQOL, and there is no way to know whether, if they indeed existed in MILES, true differences between treatment and placebo arms in these two domains would have been detected by ATAQ-LAM.

The fact that the Cough domain and Emotional Well-being scores did not differ between subjects with the lowest—versus highest—FEV1% values is not terribly surprising: with only 17 subjects with useable FEV1 data (spirometry performed within three months of completing the questionnaire), we may simply have lacked power to detect such a relationship between cough and FEV1 if, indeed, one truly exists. More likely is that cough is independent of FEV1% in LAM. The same may be true for emotional well-being—there are a number of factors besides FEV1 that could contribute to a person’s sense of well-being.

There are limitations to this work that is but the first of many more needed to build confidence in ATAQ-LAM. The number of respondents was low; however, for a rare disease (LAM affects maybe 5000-10,000 people in the U.S.), 69 respondents is a relatively large proportion of patients. Even so, there were not enough subjects to allow us to perform a reliable factor analysis. Respondents completed the questionnaire at LAMposium which may have affected responses—traveling to Chicago for the meeting could have been tiring and/or stressful; seeing friends (other patients) for the first time in a long time could have “artificially” increased patients’ moods (and thus HRQL). For the FEV1% analyses, only 17 subjects had spirometry within three months of completing the questionnaire. In future studies, it will be helpful to have subjects complete the questionnaire prior to completing spirometry on the same day—or at least within the same week. Moving forward, it will also be interesting and informative to administer ATAQ-LAM alongside other patient-reported outcome measures (e.g., SF-36) to compare and contrast scores and their implications.

Despite these limitations, we have generated a multi-dimensional instrument whose items have convergent and discriminant validity. The instrument possesses excellent face validity, appears to have acceptable content validity, and is able to differentiate patients hypothesized to have differing degrees of HRQL impairment. Through future work, the validation process will continue (e.g., by determining the ability of ATAQ-LAM scores to capture change in HRQL over time) and estimates of the minimal important difference for its scores can be triangulated.

## Additional file

Additional file 1:
**Supplemental material.**

## References

[1] Corrin B, Liebow AA, Friedman PJ (1975). Pulmonary lymphangiomyomatosis. A review. Am J Pathol.

[2] McCormack FX, Travis WD, Colby TV, Henske EP, Moss J (2012). Lymphangioleiomyomatosis: calling it what it is: a low-grade, destructive, metastasizing neoplasm. Am J Respir Crit Care Med.

[3] Taylor JR, Ryu J, Colby TV, Raffin TA (1990). Lymphangioleiomyomatosis. Clinical course in 32 patients. N Engl J Med.

[4] Ryu JH, Moss J, Beck GJ, Lee JC, Brown KK, Chapman JT, Finlay GA, Olson EJ, Ruoss SJ, Maurer JR (2006). The NHLBI lymphangioleiomyomatosis registry: characteristics of 230 patients at enrollment. Am J Respir Crit Care Med.

[5] Belkin A, Albright K, Fier K, Desserich J, Swigris J (2014). “Getting stuck with LAM”: patients perspectives on living with Lymphangioleiomyomatosis. Health and Quality of Life Outcomes.

[6] McCormack FX, Inoue Y, Moss J, Singer LG, Strange C, Nakata K, Barker AF, Chapman JT, Brantly ML, Stocks JM (2011). Efficacy and safety of sirolimus in lymphangioleiomyomatosis. N Engl J Med.

[7] Guyatt G, Feeny D, Patrick D (1993). Measuring health-related quality of life. Ann Intern Med.

[8] Hays R, Fayers P, Fayers P, Hays R (2005). Evaluating multi-item scales. Assessing quality of life in clinical trials: Methods and Practice.

[9] Meng X, Rosenthal R, Rubin D (1992). Comparing correlated correlation coefficients. Psychol Bull.

[10] Bond T, Fox C (2007). Applying the Rasch Model: Fundamental Measurement in the Human Sciences.

[11] Linacre J (2002). What do Infit and Outfit, Mean-square and Standardized mean?. Rasch Measurement Transactions.

[12] Cronbach L (1951). Coefficient alpha and the internal structure of tests. Psychometrika.

[13] Aaronson N, Alonso J, Burnam A, Lohr KN, Patrick DL, Perrin E, Stein RE (2002). Assessing health status and quality-of-life instruments: attributes and review criteria. Qual Life Res.

[14] Swigris JJ, Lee HS, Cohen M, Inoue Y, Moss J, Singer LG, Young LR, McCormack FX (2013). St. George’s Respiratory Questionnaire Has Longitudinal Construct Validity in Lymphangioleiomyomatosis. Chest.

