# Supplementary material for: Development of ATAQ-LAM: a tool to assess quality of life in Lymphangioleiomyomatosis
Source: Health Qual Life Outcomes. 2015 Jul 30;13:112. doi: 10.1186/s12955-015-0294-5 (PMC4518581; doi:10.1186/s12955-015-0294-5)
Supplement: Additional file 1: — Supplemental material. [file 12955_2015_294_MOESM1_ESM.docx]

**SUPPLEMENTAL MATERIAL**

**Development of ATAQ-LAM: A Tool to Assess Quality of Life in Lymphangioleiomyomatosis**

Tarik D. Walker, MD, MPH^1^

Jennifer Desserich, BS^2^

Karen Albright, PhD^3^

Frederick S. Wamboldt, MD^4^

Amanda Belkin, MPH^2^

Kaitlin Fier, MPH^2^

Jeffrey J. Swigris, DO, MS^2^

^1^Department of Pediatric Infectious Disease, University of Colorado School of Medicine; Aurora, Colorado

^2^Autoimmune Lung Center and Interstitial Lung Disease Program;

National Jewish Health; Denver, Colorado

^3^Department of Community and Behavioral Health, Colorado School of Public Health and Colorado Health Outcomes Program, University of Colorado School of Medicine; Aurora, Colorado

^4^Division of Pulmonary and Critical Care Medicine, Sleep and Behavioral Health Sciences Section, National Jewish Health; Denver, Colorado

During the development of this study, Dr. Swigris was supported in part by a Career Development Award from the NIH (K23 HL092227). This work was supported in part by an investigator-initiated grant from the LAM Foundation.

Author Correspondence:

Jeffrey J. Swigris, DO, MS

Associate Professor of Medicine

Autoimmune Lung Center and Interstitial Lung Disease Program

National Jewish Health

1400 Jackson Street

Denver, Colorado 80206

Phone: (303) 398-1621

Fax: (303) 398-1040

email: swigrisj@njc.org

**METHODS**

**Statistical approach**

We subjected all items in aggregate to Rasch analysis (Winsteps, Version 3.69.1.14, www.winsteps.com). First, HRQL items are calibrated on a linear scale from most likely to least likely (or easiest to most difficult) to be endorsed with the highest-value response option (a “6” in this case). Thus, items lowest on the scale connote the least amount of impairment in HRQL—items located higher on the scale are associated with greater HRQL impairment than any item(s) located lower on the scale. Similarly, the Rasch model locates patients on the same scale according to their level of HRQL impairment (as determined by their responses to all items)—patients located higher on the scale have greater HRQL impairment than any patient(s) located lower on the scale. Bond, 2007 #1665} Rasch uses the difference between item and patient location—many terms have been used, but here we refer to these locations as item difficulty and patient severity respectively—to model the probability of responses to each item. Because Rasch analysis is based on Guttman scaling, a patient’s endorsement of any item implies her endorsement with less difficult items (i.e., items lower on the scale).

Rasch analysis allows determination of whether a dataset adheres to fundamental measurement properties, an important one being unidimensionality—items function together to assess a singular construct (in the case of ATAQ-LAM, it would be LAM-specific HRQL). Thus, we used Rasch to assess the fit of the individual items as well as the items in aggregate to this unidimensional model. We assessed item fit by using the infit mean square statistic; values from 0.5-1.5 are considered useful for measurement, and values greater than 2.0 degrade measurement. [1]

**RESULTS**

**Table E1. Characteristics of 11 patients who participated in debrief**

| **Variable** | **Results** |
| --- | --- |
| Female | 11 |
| Age in years | 55.5±11.2 (range 36 – 68) |
| Race^b^  African-American  Asian  Hispanic  White | 0  0  1  10 |
| Education^c^  Some college  Associates degree  College graduate  Masters | 1  2  4  4 |
| Employment^d^  Full-time  Part-time  Retired  Disabled  Unemployed | 2  5  2  1  1 |
| Smoking history^e^  Current  Former  Never | 0  3  8 |
| LAM duration, years | 10.6±10.6(range 2 – 40) |
| FEV1, L | 1.4±0.4 (range 0.78 – 2.21) |
| FEV1% | 50.8±17.2 (range 33 – 75) |
| Supplemental oxygen^e^  Never  Ever | 4  7 |
| Medications  Combination CS/LABA  doxycycline  plaquenil  rapamycin  statin | 6  0  0  9  3 |

Footnote: LAM=lymphangioleiomyomatosis; FEV1%=percent predicted one-second forced expiratory volume; CS/LABA=combination inhaled corticosteroid and long-acting beta-agonist

**1. Item deletion algorithm**

A. Items deleted because fewer than five response options were used

**3. While sitting down, While sitting down,**

**relaxing, reading, or relaxing, reading,**

**watching TV, I was not or watching TV, I was**

**short of breath at all. extremely short of breath.**

**5. Grooming (e.g., Grooming (e.g.,**

**brushing my teeth, brushing my teeth,**

**fixing my hair) fixing my hair)**

**did not make me made me**

**short of breath at all. extremely short of breath.**

**11. Setting/clearing the Setting/clearing the**

**table for meals did not table for meals**

**not make me short made me**

**of breath at all. extremely short of breath.**

B. Items deleted because of missing responses from greater than 20% of the sample

**50. I felt completely secure I felt insecure in my**

**in my relationship with relationship with my**

**my partner. partner.**

**51. My libido has not My libido has been low. been low.**

C. Items with greater than 49% of respondents scoring at the floor (N=13)

**8. Walking from Walking from**

**room to room inside room to room inside**

**my home did not make my home made me**

**me short of breath at all. extremely short of breath.**

**9. Getting ready to Getting ready to**

**leave my home (e.g., leave my home (e.g.,**

**find my keys, find/put find my keys, find/put**

**on my coat, close the on my coat, close the**

**windows, lock the doors) windows, lock the doors)**

**did not make me short made me**

**of breath at all. extremely short of breath.**

**12. After eating a meal After eating a meal**

**I had no shortness I was extremely short**

**of breath. of breath.**

**20. Aches or pains in my Aches or pains in my**

**chest did not prevent chest prevented me from**

**me from doing doing things**

**anything I wanted to do. I wanted to do.**

C (cont’d). Items with greater than 49% of respondents scoring at the floor (N=13)

**22. I never coughed when I coughed every time**

**I took a deep breath. I took a deep breath.**

**24. My coughing did not My coughing embarrassed**

**embarrass me at all. me a great deal.**

**26. Coughing had no adverse Coughing left me**

**effect on my energy. completely wiped out.**

**27. Coughing never made Every time I coughed I felt**

**me short of breath. short of breath.**

**28. I never coughed when I coughed every time**

**I over-exerted myself. I over-exerted myself.**

**30. My coughing was My coughing was**

**not frightening extremely frightening**

**to me at all. to me.**

**32. I never wheezed. I wheezed frequently.**

**33. Overall, wheezing Overall, wheezing**

**was not a was an extremely big**

**problem for me. problem for me.**

**45. I did not have any I felt panicked all the**

**episodes of panic. time.**

**53. Cough has had no Cough has made my**

**adverse effect on quality of life**

**my quality of life. extremely poor.**

**NOTE: Despite the floor effects, item 53 was retained.**

D. Items deleted due to high item-item correlations

**23. I never coughed when I coughed every time**

**I was breathing hard or I was breathing hard or**

**fast. fast.**

Correlation with Item 28 = 0.78.

**35. In the last 2 days, In the last 2 days, I didn’t**

**I got a lot more done get anything done that I**

**than I thought I would. needed to get done.**

Correlation with Item 36 = 0.70 and concern over lack of specificity to LAM-related effects.

**36. I had enough I did not have enough**

**energy to do energy to do**

**all the things I any of the things I**

**liked or needed to do. liked or needed to do.**

Correlation with Item 34 = 0.83.

D (cont’d). Items deleted due to high item-item correlations

**38. Getting myself ready Getting myself ready**

**to leave the house to leave the house took an**

**took very little energy. extreme amount of energy.**

Correlation with Item 37 = 0.76.

**47. I did not feel anxious I felt anxious or “on edge”**

**or “on edge.” all the time.**

Correlation with Item 44 = 0.76.

**48. My relationships with My relationships with**

**family members have family members have**

**been completely free been full of**

**of stress or strain. of stress or strain.**

Correlation with Item 49 = 0.82.

**49. My relationships with My relationships with**

**my friends have my friends have**

**been completely free been full of**

**of stress or strain. of stress or strain.**

Correlation with Item 48 = 0.82 and concern over meaningfulness of change over time.

**2. Multi-item matrix:**

A. First iteration of the multi-trait scaling analysis with 32 retained items

We moved the 4 “Effects of dyspnea” items from their own scale (scale 2 above) to scale 1 (“Exertional dyspnea”). *=items hypothesized to belong to the scale named by the column header

**15. Shortness of breath Shortness of breath**

**did not prevent me prevented me from**

**from doing anything doing things**

**I wanted to do. I wanted to do.**

**16. Once I became short of Once I became short of**

**breath, it took no time breath, it took an extremely**

**at all for me to breathe long time for me to breathe**

**normally again. normally again.**

**17. I physically exerted I limited my physical**

**without fear exertion, because I was**

**of how short of breath fearful of becoming**

**I might become. too short of breath.**

**18. When I exerted When I exerted**

**physically, my physically, my**

**stamina stamina**

**was excellent. was extremely poor.**

B. Here is the resulting matrix.

Next, we moved the three items adjacent to the red arrows from the “Symptom-specific HRQL” scale (scale5) to their respective better-fitting scales. The matrix presented in the manuscript is the one resulting after moving the three items in scale 5 (items 27, 28, 29) as depicted.

This item…

**52. Shortness of breath Shortness of breath**

**has had no adverse has made my**

**effect on my quality of life**

**quality of life. extremely poor.**

was moved to the “Exertional dyspnea” scale.

This item…

**53. Cough has had no Cough has made my**

**adverse effect on quality of life**

**my quality of life. extremely poor.**

was moved to the “Cough” scale.

This item…

**54. My energy level/fatigue My energy level/fatigue**

**has had no adverse has made my**

**effect on my quality of life**

**quality of life. extremely poor.**

was moved to the “Fatigue” scale.

These items (the remaining two items from scale5 and the lone item from scale6) were retained and contribute to a total score but are not scored in any of the scales.

**19. I have had no I have had severe**

**aches or pains in aches or pains in**

**my chest. my chest.**

**55. Despite having LAM Because of LAM**

**my day-to-day life has my day-to-day life has**

**been free of hassles been full of hassles and**

**or inconveniences. inconveniences.**

**56. My quality of life My quality of life**

**has been excellent. has been extremely poor.**

3. Results of the exploratory factor analysis.

**Table E2.** **Eigenvalues and cumulative variance explained.**

| **Eigenvalues of the Correlation Matrix: Total = 4 Average = 1** | | | | |
| --- | --- | --- | --- | --- |
|  | **Eigenvalue** | **Difference** | **Proportion** | **Cumulative** |
| **1** | 2.88731437 | 2.28825785 | 0.7218 | 0.7218 |
| **2** | 0.59905652 | 0.25245146 | 0.1498 | 0.8716 |
| **3** | 0.34660506 | 0.17958101 | 0.0867 | 0.9582 |
| **4** | 0.16702405 |  | 0.0418 | 1.0000 |

**Figure E1. Scree plot from the EFA.**

**Table E3. Factor pattern from the EFA.**

| **Factor Pattern** | |
| --- | --- |
|  | **Factor1** |
| **SCALE1** | 0.86717 |
| **SCALE2** | 0.75834 |
| **SCALE3** | 0.90707 |
| **SCALE4** | 0.85876 |

**Table E4. Final communality estimates from the EFA.**

| **Final Communality Estimates: Total = 2.887314** | | | |
| --- | --- | --- | --- |
| **SCALE1** | **SCALE2** | **SCALE3** | **SCALE4** |
| 0.75198923 | 0.57507632 | 0.82278160 | 0.73746723 |

**REFERENCES**

1. Linacre J: **What do Infit and Outfit, Mean-square and Standardized mean?** *Rasch Measurement Transactions* 2002, **16:**878.
